# Digital Competence Among Older Adults: Is Self-Assessment Reliable?

**DOI:** 10.7759/cureus.73543

**Published:** 2024-11-12

**Authors:** Mircea-Dan Marzan, Andreea Stamate, Luiza Spiru

**Affiliations:** 1 Geriatrics, Carol Davila University of Medicine and Pharmacy, Bucharest, ROU; 2 Research, Ana Aslan International Foundation, Bucharest, ROU; 3 Geriatrics and Gerontology, Ana Aslan International Foundation, Bucharest, ROU

**Keywords:** digital competence, digital technology, objective evaluation, older adults, self-assessment

## Abstract

Introduction: Digital technology holds promise for addressing age-related challenges such as diminished physical and cognitive abilities, chronic conditions, and shifts in social connections. Individual proficiency in computer and internet skills influences technology adoption, correlated with advanced usage, positive attitudes, and self-efficacy. Despite extensive research on digital competence among younger demographics, there is a noticeable gap in inclusive design and research for older adults, with existing assessments often overlooking user ability diversity. The aim of this study is to evaluate the reliability of self-assessment as a method for measuring digital skills among older adults by comparing self-reported competence with objective assessments using the digital foundation skills framework.

Methods:A between-subjects design was used to explore the reliability of self-assessment in evaluating digital skills among older adults, comparing self-assessed and objective assessments using the digital foundation skills framework. This framework encompasses six domains and provides statements and practical examples for self-assessment and objective evaluation of digital competence. Data were collected from 51 Romanian adults over the age of 60 using an online survey for the subjective assessment and laboratory testing for the objective assessment.

Results:Significant disparities were found between self-assessed and actual performance in three domains: digital foundation skills, communicating, and problem-solving. However, older adults accurately assessed their performance in handling information and content, transacting, and being safe and legal online.

Conclusion:Given the lack of a consistent pattern of overestimation or underestimation, objective assessment is recommended for precise digital competence evaluation.

## Introduction

In the context of global ageing, digital technology is seen as a promising solution to address challenges linked to ageing, such as diminished physical and cognitive function, multiple chronic conditions, and changes in social connections [1]. The potential of digital technology in promoting healthy ageing has been recognised in various studies, highlighting its role in maintaining independence [2], preventing injuries [3], enhancing social connectedness [4], and contributing to mental health treatment [5]. Prior studies have shown that the utilisation of computers and the internet among older individuals facilitates communication [6,7], information and learning and staying updated [8], enhances performance in daily tasks such as shopping and banking [9], grants access to health-related information [10], and enriches social interactions [11]. Moreover, these technologies can serve as compensatory tools for memory lapses and cognitive decline [2]. However, the extent of the benefits derived from these technologies is closely tied to the user's computer proficiency [12].

Computer and internet usage may hinge on individual computer and internet skills, with research suggesting that a lack thereof can act as a barrier to adopting new technologies [13]. Proficiency in computer skills has been positively correlated with the adoption and use of advanced technologies [14,15], favourable attitudes towards computers, and higher computer self-efficacy [16]. Lack of proficiency may facilitate computer anxiety [17,18]. Morrell et al. [19] found that a lack of interest in the internet and internet usage was associated with the inability to use the internet. Furthermore, computer proficiency has been identified as a predictor for sustained engagement with digital technologies over time [20]. Therefore, facilitating efficient and effective training to increase older adults' computer proficiency is a pivotal objective in order to ensure that they can reap the multiple benefits of digital technologies. A crucial precondition for achieving this goal is understanding the initial levels of computer proficiency among older adults [15]. Additionally, comprehending how older adults self-assess their abilities is essential, as research in other domains suggests that either underestimating or overestimating one's capabilities can impact learning outcomes [21]. For instance, according to Bandura [22], individuals lacking confidence in their abilities are less likely to engage in tasks and more prone to giving up in the face of challenges, while a slight overestimation of abilities tends to boost effort and persistence.

Understanding the digital competence of older adults is also crucial in the context of digital cognitive testing. An issue in computerised cognitive assessment is that varying levels of computer experience may lead to performance discrepancies on cognitive tests, regardless of actual cognitive status. Relying on computerised tests for clinical decision-making could result in overdiagnosis among individuals with limited computer experience and underdiagnosis among those with extensive computer experience [23]. Knowledge of older adults' computer proficiency is also vital in designing new applications. Taking into account the unique needs and constraints of older adults in the design of forthcoming digital technologies could ensure an enhanced user experience. This consideration has the potential to advance technologies for assessment and diagnosis, aiming to maximise patient benefits while mitigating potential risks [24].

As digital technology emerges as a promising solution to confront the multifaceted challenges associated with ageing, it becomes imperative to explore the digital competence of older adults. Despite numerous studies assessing the digital literacy of younger generations, there is a notable gap in research and design inclusivity for older adults in digital technologies [25]. Additionally, prevalent measures of digital competences for older adults primarily concentrate on acceptance models and adoption barriers, neglecting user ability heterogeneity [25]. The initial step in supporting older adults to harness the digitalisation trend in healthcare is accurately measuring their digital competences [25].

Digital competence is a vital skill for lifelong learning in our increasingly digitalised society. It encompasses various terms such as information and communications technology (ICT) skills, technology skills, information technology skills, 21st-century skills, information literacy, digital literacy, and digital skills. These terms are often used interchangeably [26]. Jenkins [27] highlighted 21st-century literacy, focusing on social skills. The diverse terminology reflects rapid technological advancements and different areas of interest. Changes in society and culture influence these terms. Ala-Mutka et al. [28] recommended dynamic and regular revisions due to evolving technologies. In the widest and most recent definitions, based on policy-related papers and reports, digital competence consists not only of digital skills but also of social and emotional aspects of using and understanding digital devices [29]. The European Commission [29] has defined digital competence as involving the confident and critical use of Information Society Technology for work, leisure, and communication. Digital competence is grounded on basic skills in ICT, i.e., the use of computers to retrieve, assess, store, produce, present and exchange information, and to communicate and participate in collaborative networks via the internet [30].

The majority of studies assessing the digital competence of older adults have concentrated on aspects such as electronic health, technology acceptance, and attitudes. A recent systematic review has found that the most frequently utilised instrument, accounting for 59% of the studies included in their review, was the eHealth Literacy Scale [25]. Other studies employed the Unified Theory of Acceptance and Usage of Technology (e.g., [31]) and Loyd-Gressard's Computer Attitude Scale (e.g., [32]), with varying focuses on computer anxiety and confidence. Notably, there is a dearth of research that compares self-report to actual digital competence. In research studies, self-reported measures are frequently used to gauge individuals' perceptions of their digital competence. Nevertheless, when researchers objectively evaluate participants' digital abilities, differences between self-reported and actual skills may emerge.

As previously stated, it is crucial to account for the diverse capacities of older adults and measure literacy elements applicable to the broader population, covering communication, safety, problem-solving, and competence [25]. According to Hänninen et al. [33], the digital capacity of older adults spans a continuum from actively independent to limited, underscoring the necessity for nuanced assessments of their digital competences. In light of this need for tailored evaluations, the objective of the study is to assess the reliability of self-assessment as a method for measuring digital skills among older adults. This will be achieved by comparing self-reported competence with objective assessments utilising the digital foundation skills framework. The study seeks to address the existing research gap regarding digital competence in older adults, emphasising the importance of inclusive design and the consideration of diverse user abilities in digital skill assessment.

## Materials and methods

Transparency and openness

Neither the design, hypotheses, nor analytic plan for this study were preregistered. The supplementary materials, deidentified data, and SPSS syntax that support the findings of this study can be accessed at https://osf.io/pskg8/?view_only=ff4f385681a8480fb8d8e07e59fb053a.

Participants

In Romania, the use of digital health tools among older adults is shaped by a range of socio-cultural and sociolinguistic factors that provide essential context for understanding their engagement with such technologies. Romanian is the primary language, and most healthcare interactions occur in Romanian. However, literacy levels vary widely, especially among older adults from rural areas or those with limited formal education, impacting their comprehension of health information or digital instructions. Additionally, Romania is home to regions where Hungarian and Saxon (German) languages are prevalent, meaning that older adults in these areas may be more comfortable accessing health information and services in their native languages. This language diversity adds another layer of complexity to digital health adoption, as language compatibility can be a barrier to the effective use of technology.

Culturally, traditional healthcare practices are highly trusted, with older adults often favouring face-to-face consultations over digital solutions. Family support is central, so older adults tend to rely on family members for assistance rather than digital tools, especially given their limited familiarity with technology. Generational gaps also impact comfort with digital devices, as many grew up with little exposure to modern technology and, therefore, prefer direct, human guidance. Additionally, economic constraints, such as low pensions, limit technology access, particularly in rural or lower-income areas, which can restrict older adults' ability to purchase or maintain digital devices. Together, these socio-cultural, linguistic, and economic factors significantly influence the feasibility and acceptance of digital health tools among Romania's older adult population.

A convenience sample of 51 participants was recruited from Centrul de Excelență pentru Boli de Memorie și Medicina Longevității clinic in Bucharest, Romania, by means of purposive sampling. All participants were selected by the clinics' geriatricians from their patient pool. The eligibility requirements encompassed individuals aged 60 years or older, residing independently at home with access to the internet, familiarity with ICT such as tablets, smartphones, or computers, and the absence of acute or severe illnesses, such as dementia, or scheduled surgeries. Additional exclusion criteria involved self-reported hearing or vision impairments significantly affecting daily functioning and reliance on assistive devices for walking, ensuring participants' ability to actively engage in both self-assessment (via online survey) and objective evaluation (laboratory testing). All participants provided written consent. All participants were Caucasian Europeans (Romanians). The statements were translated into Romanian by the second author, who has completed her higher education, including her PhD, in English and is a native Romanian speaker.

Design

The experiment employed a within-subjects design. The participants took part in two evaluations. The first evaluation was conducted online. Specifically, participants had to fill in a self-assessment form evaluating their digital competence. For the second evaluation, the objective assessment of their digital competence, the participants were tested individually in the laboratory. Both for self-assessment and objective assessment, we used the digital foundation skills framework [34]. The framework was designed for use by all individuals across the United Kingdom (UK) who are engaged in assisting adults to enhance their fundamental digital competencies. The Tech Partnership and Lloyds Banking Group led the coordination of the consultation and revision process for the framework. This effort was supervised by a steering committee comprised of representatives such as the Department for Education, Department for Digital, Culture, Media & Sport, Department for Work and Pensions, Microsoft, and NHS Digital, to name a few.

The digital foundation skills framework comprises six domains: digital foundation skills, communicating online, handling information and content, transacting online, problem-solving, and being safe and legal online. This framework provides statements such as "I can use word processing applications to create documents" along with practical examples such as "Use word processing software to create a curriculum vitae (CV) or a letter". This dual approach enabled us to utilise the framework for both self-assessment of digital competence and objective evaluation (see the Appendices section for all the items).

For the self-assessment, we developed a Qualtrics survey incorporating the framework's statements. The survey included three response options: "I can perform the task alone", "With guidance", and "Not at all". The participants completed the survey using their personal devices (mobile phones or computers).

In the objective evaluation, we utilised the framework's practical examples. The researcher instructed the participant to carry out specific actions based on these examples, for instance, "Please use a word processing software to create a CV or a letter". The researcher then marked whether the participant completed the task independently, with guidance (limited to one hint from the researcher), or not at all. The participants underwent testing on a PC with a keyboard and mouse for most tasks, except for tasks related to digital foundation skills (subtask 1.5) and transacting (subtask 4.2), which were conducted on a tablet.

For the purpose of the analysis, we allocated 2 points for performing a task independently, 1 point for completing the task with guidance, and 0 points for not being able to perform the task. Several variables were constructed to facilitate this analysis: "self-assessed general digital competence" (SAGDC), which represents the sum of self-reported scores from the total of 44 items of the digital foundation skills framework; and "objective general digital competence" (OAGDC), which reflects scores obtained from the same 44 items during the objective assessment.

Additionally, we calculated both self-assessed and objective competence for each of the six domains of the framework, resulting in 12 variables: Objective Assessment of Digital Foundation Skills (OADFS), Self-Assessment of Digital Foundation Skills (SADFS), Objective Assessment of Communicating Online (OACO), Self-Assessment of Communicating Online (SACO), Objective Assessment of Handling Information and Content (OAHIC), Self-Assessment of Handling Information and Content (SAHIC), Objective Assessment of Transacting Online (OATO), Self-Assessment of Transacting Online (SATO), Objective Assessment of Problem-Solving (OAPS), Self-Assessment of Problem-Solving (SAPS), Objective Assessment of Being Safe and Legal Online (OASLO), and Self-Assessment of Being Safe and Legal Online (SASLO). The cognitive status of each participant was assessed using the Montreal Cognitive Assessment scale (MoCA), and testing was conducted in Romanian, which is the native language of the researcher and all participants.

An a priori power analysis was conducted using G*Power version 3.1.9.7 (Heinrich Heine University Düsseldorf, Düsseldorf, Germany) for sample size estimation. The effect size in the study was 0.5, which is considered medium using Cohen's criteria. With a significance criterion of α = .05 and power = .80, the minimum sample size needed with this effect size is N = 33 for a paired-sample t-test, which will be used to test the hypothesis. Thus, the obtained sample size of N = 51 is more than adequate to test the study hypothesis.

Data analysis

Data were analysed using IBM SPSS Statistics for Windows, Version 25 (Released 2017; IBM Corp., Armonk, New York). Statistical significance was set at alpha = .05. The differences between older adults' self-assessed scores and their objective scores for general digital competence, as well as for each of the six domains of the digital foundation skills framework [34], were analysed using Wilcoxon signed-rank tests. The correlations between SAGDC scores, objective digital competence scores, age, level of education, and cognitive status (MoCA scores) were analysed using Spearman correlations. A multiple regression was run to predict objective digital competence from age and MoCA scores.

## Results

The participant demographic data are presented in Table 1.

**Table 1:** Characteristics of participants at baseline MoCA: Montreal Cognitive Assessment

Characteristic	N	Min.	Max.	Mean	Std.
Age (years)	51	60.00	80.00	71.90	5.18
Education (years)	51	8.00	16.00	13.71	2.02
MoCA	51	20.00	29.00	25.14	2.13
Female	44 (86.3%)	-			
Male	7 (13.7%)	-			

Skewness, kurtosis, and normality tests for general digital competence, age, education, and cognitive status are presented in Table 2. Digital competence scores, as assessed by Shapiro-Wilk's test, were normally distributed for objective general digital competence and for age in years but not for SAGDC, number of years of formal education, and cognitive status (MoCA score). The means and standard deviations for general digital competence and digital competence for each subdomain of the DigComp [35], in scores, are presented in Table 3.

**Table 2:** Skewness, kurtosis, and normality tests for age, education, cognitive status (MoCA score) objective and self-assessed general digital competence scores *indicates a statistically significant deviation from normal distribution SE: standard error

Variable	Skewness			Kurtosis			Shapiro-Wilk Test
	Value	SE	Z	Value	SE	Z	p-value
Age (in years)	-0.444	0.333	-1.33	-0.467	0.656	-0.71	0.085
Education (in years)	-0.395	0.333	-1.18	-0.455	0.656	-0.60	<0.001*
Cognitive status	-0.226	0.333	-0.67	0.420	0.656	0.64	0.009*
Objective digital competence	0.310	0.333	0.93	-0.403	0.656	-0.61	0.186
Self-assessed digital competence	-0.334	0.333	-1.00	-1.143	0.656	-1.74	0.004*

**Table 3:** Descriptive statistics for digital competence scores M: mean; SD: standard deviation

Type of Assessment and Domain	Score M	Score SD
Objective assessment general digital competence	44.98	18.21
Self-assessment general digital competence	52.45	27.26
Objective assessment digital foundation skills	7.90	3.66
Self-assessment digital foundation skills	10.06	3.00
Objective assessment communicating online	6.49	3.06
Self-assessment communicating online	8.41	3.72
Objective assessment handling information and content	8.76	3.71
Self-assessment handling information and content	9.45	5.68
Objective assessment transacting online	5.59	3.73
Self-assessment transacting online	6.43	4.48
Objective assessment problem-solving	5.59	3.73
Self-assessment problem-solving	3.47	2.21
Objective assessment being safe and legal online	13.86	5.47
Self-assessment being safe and legal online	14.63	10.72

Correlations between age, education, cognitive status, and digital competence

Spearman correlation results showed that there was a significant negative relationship between age and objective digital competence scores r(49) = -.50, p < .001, as well as between age and SAGDC r(49) = -.40, p = .003. There was also a statistically significant positive relationship between cognitive status (MoCA scores) and objective general digital competence scores r(49) = .48, p <.001, as well as SAGDC scores r(49) = .31, p = .028. There was a significant positive relationship between objective digital competence scores and age SAGDC r(49) = .76, p < .001 (Table 4).

**Table 4:** Descriptive statistics and correlations for age (in years), education (in years), cognitive status (MoCA score), Self-Assessed General Digital Competence (SAGDC scores), and Objective General Digital Competence (OAGDC scores) *indicates a statistically significant relationship at p < .05 **indicates a statistically significant relationship at p < .01

Variable	n	M	SD	1	2	3	4
1. Age	51	71.9	5.18	N/A	N/A	N/A	N/A
2. Education	51	13.71	2.02	0.03	N/A	N/A	N/A
3. Cognitive status (MoCA score)	51	25.14	2.13	-0.09	0.16	N/A	N/A
4. Self-assessed general digital competence	51	52.45	27.26	-.40**	-0.05	.31*	N/A
5. Objective general digital competence	51	44.98	18.21	-.50**	0.04	.48**	.76**

Comparison between self-assessed and objective digital competence

Wilcoxon signed-rank tests indicated that older adults' SAGDC scores were significantly higher than their objective general digital competence scores. Statistically significant differences between self-assessed scores and objective scores were found in the digital foundation skills, communicating, and problem-solving subdomains. In the remaining three subdomains, handling information and content, transacting online, and being safe and legal online, there were no statistically significant differences between self-assessed and objective digital competence (Table 5).

**Table 5:** Results of the Wilcoxon signed-rank test—comparison between self-assessed and objective digital competence scores (general and for each of the six competence subdomains) *indicates a statistically significant difference at p < .01 ^b^Based on positive ranks ^c^Based on negative ranks OAGDC-SAGDC: Objective assessment general digital competence - Self-assessment general digital competence; OADFS-SADFS: Objective assessment Digital Foundation skills - Self-assessment Digital Foundation; OACO-SACO: Objective assessment Communicating online - Self-assessment Communicating online; OAHIC-SAHIC: Objective assessment Handling information and content - Self-assessment Handling information and content; OATO-SATO: Objective assessment Transacting online - Self-assessment Transacting online; OAPS-SAPS; Objective assessment Problem-Solving - Self-assessment Problem-Solving; OASLO-SASLO: Objective assessment Being safe and legal online - Self-assessment Being safe and legal online

Variable	N	Mean Rank	Sum of Ranks	N	Mean Rank	Sum of Ranks	Ties	Z	p-value
OAGDC-SAGDC	33	27.86	919.5	17	20.91	355.5	1	-2.723^b^	.006*
OADFS-SADFS	33	22.65	747.5	8	14.19	113.5	10	-4.124^b^	< .001*
OACO-SACO	32	22.39	716.5	9	16.06	144.5	10	-3.719^b^	< .001*
OAHIC-SAHIC	29	21.74	630.5	17	26.5	450.5	5	-.986^b^	0.324
OATO-SATO	22	24.93	548.5	19	16.45	312.5	10	-1.535^b^	0.125
OAPS-SAPS	11	12.64	139	32	25.22	807	8	-4.052^c^	< .001*
OASLO-SASLO	27	25.06	676.5	22	24.93	548.5	2	-.637^b^	0.524

Age and cognitive status as predictors of objective digital competence

We employed a multiple linear regression model to assess the contributions of age and cognitive status on objective general digital competence. The model is specified as Y = β0 + β1X1 + β2X2 + ε. The overall fit of the model was statistically significant, F(2,48) = 16.312, p < .001, suggesting that the model explains a significant portion of the variance in objective general digital competence. The adjusted R^2^ value of .38 further illustrates that the model can account for approximately 38% of the objective general digital competence scores. The results from the multiple regression indicated that age negatively predicted objective general digital competence; for each additional year of age, there was a 1.57 decrease in objective general digital competence score (B= -.447, p < .001), while cognitive status positively predicted objective general digital competence score (B=.414, p = .001); for each 1 point increase in MoCA score, there was a 3.45 increase in objective general digital competence score (Table 6).

**Table 6:** Regression coefficients of age and cognitive status (MoCA scores) on objective general digital competence CI: confidence interval; SE: standard error; MoCA: Montreal Cognitive Assessment scale

Variables	B	SE	β	t	p-value	95% CI
(Constant)	68.775	38.81	-	1.77	.083	(-9.27, 146.82)
Age (years)	-1.57	.39	-.447	-3.99	.000	(-2.36, -0.78)
Cognitive status (MoCA score)	3.54	.96	.414	3.7	.001	(1.62, 5.47)

## Discussion

Based on our observational findings from pilot studies involving older adults testing different digital applications, it became apparent that self-reported digital proficiency may not accurately reflect the true digital capabilities within this demographic. In the current study, we compared self-reported digital competence, as measured with DigComp, with objective digital competence on each item of the scale in a sample of 51 older Romanian adults. We found statistically significant differences between self-reported and objective performance, with a majority of older adults overestimating their digital competence. Performance on both measures of digital competence was very heterogeneous in our sample population. There were large individual differences in digital competence as measured by both objective digital competence and self-report, as well as in individual differences between perceived and actual digital competence. We also discovered that discrepancies between self-assessed and objectively assessed competence only emerged in three out of the six domains outlined in the DigComp. Hence, the accuracy in estimating digital competence not only varies among individuals but also varies at the individual level across different domains of digital competence, further emphasising the vulnerability of self-evaluation.

Our results align with prior research conducted on younger individuals. McCourt et al. [36] conducted a comparison between self-reported and actual proficiency across six areas of computer literacy (general IT awareness, spreadsheets, word processing, databases, email/internet, and presentation) among 382 undergraduate students from two UK universities who assessed their own proficiency levels. While they employed different instruments and methods for self-assessment and objective assessment, their results also revealed noteworthy distinctions between students' perceived and actual computer literacy levels, with most students overestimating their proficiency. In their study, students with higher abilities tended to provide more accurate self-assessments. Based on their findings, the authors proposed that relying solely on self-assessment is inadequate for assessing computer literacy among entry-level undergraduate accounting students. Our results also indicate that older adults are not homogenous in their estimation of their digital competence, thus questioning the reliability of self-assessment measures.

Similarly, Merrit et al. [37] found significant gaps between self-reported measures of computer literacy and objective measures of computer literacy assessed using SAM Challenge (a computer-based computer literacy test) in a sample of 55 students. The authors concluded that self-reported computer literacy is unreliable and proposed that this fact has serious implications in several areas: in research, when self-reported computer literacy is used as a variable, in educational settings, and in businesses that need to employ candidates with a certain level of computer literacy.

Evidence for people's tendency to overestimate their digital skills also comes from the grey literature. The paper "Perception and Reality: Measuring Digital Skills in Europe" presents the main findings of studies conducted in five digitally advanced European countries measuring self-reported and actual digital skills in large samples of younger and middle-aged adults [38]. The participants assessed their digital skills in European Computer Driving Licence Base modules, which included Computer Essentials, Online Essentials, Word Processing, and Spreadsheets. Subsequently, they tackled practical questions and assignments in a SOFIA simulated work environment (an automated solution designed to assess general or specific digital skills in a testing environment resembling real-world situations). Their main findings were consistent across the five countries: self-assessment proved unreliable in predicting actual performance, with individuals often overestimating their skills; despite being digitally advanced compared to many European and global counterparts, all surveyed countries exhibited digital skills gaps that persisted across all ages.

Similar to this report, we found significant individual differences in actual digital competence among older adults. The level of digital competence was positively associated with cognitive status as measured with the MoCA scale and negatively associated with age. Similar findings were reported by Zhang et al. [12]. In a sample of 97 older adults, they found that age, cognitive factors, and education predicted computer proficiency as measured with the Computer Proficiency Questionnaire. While the positive correlation between different constructs of digital competence and education is reported in the literature, in our sample, digital competence did not correlate with education level. One possible reason is that there is minimal variation in education level among our participants, with most of them (78%) having higher education. Although this aspect poses a limitation to our study, our primary objective was to examine the accuracy of older adults' self-assessment of their digital competence rather than to identify predictors of digital competence.

Most of the studies comparing self-assessed and actual performance in different constructs related to the use of digital technologies typically involve younger adults. In two consecutive studies, Marquie et al. [39] found that, compared to younger adults, older participants underestimate their knowledge in the computer domain but not in the general culture domain. In our study, the majority of the participants exhibited overconfidence in their digital proficiency. There are several notable differences between our study and theirs that may account for the divergent results. First, Marquie et al. [39] aimed to identify how confident older adults are in their computer knowledge compared to younger adults. Our purpose was to determine how accurate older adults are in estimating their digital competence. The authors observed that older adults often underestimate their abilities, but their study focused on comparing self-efficacy beliefs between young and older adults rather than comparing these beliefs to an objective measure of computer knowledge.

In contrast, our study involved comparing older adults' self-reported performance with their actual performance measured through laboratory assessment. An important aspect of our research is the assessment of objective digital competence, where older adults completed tasks under direct supervision. It is noteworthy that older adults' performance in using digital technologies is influenced not only by knowledge but also by physical limitations, which can become apparent during task performance [40]. This assessment method also allowed us to evaluate whether individuals unable to complete a task independently could do so with assistance. This is especially relevant for telemedicine applications, where the advantage lies in the absence of the medical professional but with the possibility of a caregiver aiding seniors in task completion.

It is essential to consider that individual differences in how accurately older adults estimate their digital competence do exist, and not all older adults overestimate their digital skills. Some older individuals may accurately assess their abilities, while others may underestimate them. Furthermore, our study showed that self-assessment accuracy varies even at the individual level, depending on the digital competence domain. We found statistically significant differences between self-assessed and actual performance in only three domains: digital foundation skills, communicating, and problem-solving. In the remaining three domains (handling information and content, transacting, and being safe and legal online), older adults were able to estimate their performance accurately. Since we cannot generalise a particular type of behaviour (overestimation/underestimation) in older adults' assessment of their digital competence that would allow us to compensate for the discrepancies between self-reported and objective measures, we propose that in instances where an accurate measure of digital competence is essential, objective assessment should be used.

Limitations

Our study possesses several limitations. Firstly, we utilised a convenience sample, which did not ensure adequate variation in education levels, given that the majority of our subjects (78%) were educated at high school and graduate levels. Additionally, our sample comprised a disproportionate number of female subjects. As a result, our findings may not be generalisable to populations with lower levels of education or to the male population. Furthermore, although the sample size for our study provided sufficient statistical power, a larger sample would have allowed us to assess subgroups with different levels of experience to explore whether other factors, such as the users' actual level of digital competence, may influence self-estimation accuracy.

Implications

Our findings have implications across various domains. Firstly, they impact industries involved in developing digital applications for older adults. The decision to adopt a specific digital application is frequently swayed not only by its effectiveness but also by how user-friendly the interface is perceived by the end-user [41,24]. The characteristics that enhance usability for certain users may impede it for others. Enhancing the usage of an information system can be achieved by customising the user interface to match the digital competence level of the end-users [42]. Our findings suggest that self-assessment may not accurately reflect actual digital competence. Therefore, accurately assessing the user's computer literacy level is essential for designing improved user interfaces and consequently fostering better adoption by the end-users [42].

With the proliferation of digital applications meant to improve older adults' level of independent living and well-being or to measure different health parameters, there is also an increase in the number of studies investigating correlations between digital competence and the desired benefits of these applications. Several recent studies have investigated the relationship between digital competence and psychological well-being [43], burnout [44], stress [45], and life satisfaction [46], to name a few. In line with previous studies on younger adults, our study shows that self-reported digital competence is not reliable and may not always reflect the actual competence of older adults. Therefore, future studies need to consider objective measures when including digital competence as a variable.

## Conclusions

In conclusion, our study highlights significant discrepancies between self-reported and objectively measured digital competence among older adults, particularly in certain domains of the DigComp framework. While older adults generally overestimate their digital abilities, this misjudgement is not uniform across all digital skills. Our findings align with previous research involving younger populations, suggesting that self-assessment is an unreliable measure of digital competence. Given the individual variability and domain-specific differences in self-assessment accuracy, we recommend using objective assessments when precise digital competence is required, particularly in contexts such as telemedicine and digital inclusion initiatives for older adults.

## Appendices

Data availability statement

The de-identified data, supplementary materials, and SPSS syntax that support the findings of this study can be accessed at https://osf.io/pskg8/?view_only=ff4f385681a8480fb8d8e07e59fb053a.

## References

[1] Maharani A, Pendleton N, Leroi I (2019). Hearing impairment, loneliness, social isolation, and cognitive function: longitudinal analysis using English longitudinal study on ageing. Am J Geriatr Psychiatry.

[2] Charness N, Boot WR (2009). Aging and information technology use: potential and barriers. Curr Dir Psychol Sci.

[3] Tamura T, Yoshimura T, Sekine M, Uchida M, Tanaka O (2009). A wearable airbag to prevent fall injuries. IEEE Trans Inf Technol Biomed.

[4] Kluck JP, Stoyanova F, Krämer NC (2021). Putting the social back into physical distancing: the role of digital connections in a pandemic crisis. Int J Psychol.

[5] Fairburn CG, Patel V (2017). The impact of digital technology on psychological treatments and their dissemination. Behav Res Ther.

[6] Braun MT (2013). Obstacles to social networking website use among older adults. Comput Hum Behav.

[7] Vroman KG, Arthanat S, Lysack C (2015). "Who over 65 is online?" Older adults' dispositions toward information communication technology. Comput Hum Behav.

[8] Morris A, Goodman J, Brading H (2007). Internet use and non-use: views of older users. Univers Access Inf Soc.

[9] Wagner N, Hassanein K, Head M (2010). Computer use by older adults: a multi-disciplinary review. Comput Hum Behav.

[10] Mitzner TL, Fausset CB, Boron JB (2008). Older adults' training preferences for learning to use technology. Proc Hum Factors Ergon Soc Annu Meet.

[11] Leist AK (2013). Social media use of older adults: a mini-review. Gerontology.

[12] Zhang S, Grenhart WC, McLaughlin AC, Allaire JC (2017). Predicting computer proficiency in older adults. Comput Hum Behav.

[13] Gibbs SF (2008). Internet use equals computer literacy?. Proceedings ascilite Melbourne.

[14] Sengpiel M, Dittberner D (2008). The computer literacy scale (CLS) for older adults-development and validation. Mensch und Computer 2008.

[15] Boot WR, Charness N, Czaja SJ (2015). Computer proficiency questionnaire: assessing low and high computer proficient seniors. Gerontologist.

[16] Fagan MH, Neill S, Wooldridge BR (2004). An empirical investigation into the relationship between computer self-efficacy, anxiety, experience, support and usage. J Comput Inf Syst.

[17] Sengpiel M, Jochems N (2015). Validation of the computer literacy scale (CLS). Human Aspects of IT for the Aged Population. Design for Aging. ITAP 2015.

[18] Schlebusch CL (2018). Computer anxiety, computer self-efficacy and attitudes towards the internet of first year students at a South African University of Technology. Afr Educ Rev.

[19] Morrell RW, Mayhorn CB, Bennett J (2000). A survey of World Wide Web use in middle-aged and older adults. Hum Factors.

[20] Mitzner TL, Savla J, Boot WR, Sharit J, Charness N, Czaja SJ, Rogers WA (2019). Technology adoption by older adults: findings from the PRISM trial. Gerontologist.

[21] Janis IL, Mann L (1977). Decision making: a psychological analysis of conflict, choice, and commitment. Free press. APA.

[22] Bandura A (1986). Social foundations of thought and action. Englewood Cliffs.

[23] Lee Meeuw Kjoe PR, Agelink van Rentergem JA, Vermeulen IE, Schagen SB (2021). How to correct for computer experience in online cognitive testing?. Assessment.

[24] Robillard JM, Lai JA, Wu JM, Feng TL, Hayden S (2018). Patient perspectives of the experience of a computerized cognitive assessment in a clinical setting. Alzheimers Dement (N Y).

[25] Oh SS, Kim KA, Kim M, Oh J, Chu SH, Choi J (2021). Measurement of digital literacy among older adults: systematic review. J Med Internet Res.

[26] Adeyemon E (2009). Integrating digital literacies into outreach services for underserved youth populations. Ref Libr.

[27] Jenkins H (2006). Confronting the challenges of participatory culture: media education for the 21st century. An occasional paper on digital media and learning. John D. and Catherine T. MacArthur Foundation.

[28] Ala-Mutka K, Punie Y, Redecker C (2008). Digital competence for lifelong learning: policy brief.

[29] Punie Y, Zinnbauer D, Cabrera M (2006). A review of the impact of ICT on learning. Academia.

[30] Ilomäki L, Kantosalo A, Lakkala M (2011). What is digital competence?. https://tuhat.helsinki.fi/ws/portalfiles/portal/48681684/Ilom_ki_etal_2011_What_is_digital_.

[31] Lai HJ (2018). Investigating older adults' decisions to use mobile devices for learning, based on the unified theory of acceptance and use of technology. Interact Learn Environ.

[32] Chuvessiriporn S (1999). Hospitality students' attitudes and behavioral intentions toward learning and using computer technology. Interactive Learning Environments.

[33] Hänninen R, Taipale S, Luostari R (2021). Exploring heterogeneous ICT use among older adults: the warm experts’ perspective. New Media Soc.

[34] (2024). Essential digital skills framework. https://assets.publishing.service.gov.uk/media/5b9246d4e5274a4236952309/Essential_digital_skills_framework.pdf.

[35] Ferrari A, Punie Y (2013). DIGCOMP: a framework for developing and understanding digital competence in Europe. JRC Publications Repository.

[36] McCourt Larres P, Ballantine J, Whittington M (2003). Evaluating the validity of self-assessment: measuring computer literacy among entry-level undergraduates within accounting degree programmes at two UK universities. Account Educ.

[37] Merritt K, Smith D, Renzo JCD (2002). An investigation of self-reported computer literacy: is it reliable. Issues Inf Syst.

[38] (2024). Perception and reality: measuring digital skills in Europe. https://eufordigital.eu/library/perception-and-reality-measuring-digital-skills-in-europe/.

[39] Marquié JC, Jourdan-Boddaert L, Huet N (2002). Do older adults underestimate their actual computer knowledge?. Behav Inf Technol.

[40] Yazdani-Darki M, Rahemi Z, Adib-Hajbaghery M, Izadi-Avanji FS (2020). Older adults' barriers to use technology in daily life: a qualitative study. Nurs Midwifery Stud.

[41] Schroeder T, Dodds L, Georgiou A, Gewald H, Siette J (2023). Older adults and new technology: mapping review of the factors associated with older adults' intention to adopt digital technologies. JMIR Aging.

[42] Van Vliet PJ, Kletke MG, Chakraborty G (1994). The measurement of computer literacy: a comparison of self-appraisal and objective tests. Int J Hum Comput Stud.

[43] Buckingham S, Tu G, Elliott L, Poole R, Walker T, Bland E, Morrissey K (2023). Digital competence and psychological wellbeing in a social housing community: a repeated survey study. BMC Public Health.

[44] Wang X, Zhang R, Wang Z, Li T (2021). How does digital competence preserve university students' psychological well-being during the pandemic? An investigation from self-determined theory. Front Psychol.

[45] Kumpikaitė-Valiūnienė V, Aslan I, Duobienė J, Glińska E, Anandkumar V (2021). Influence of digital competence on perceived stress, burnout and well-being among students studying online during the COVID-19 lockdown: a 4-country perspective. Psychol Res Behav Manag.

[46] Bae SM (2022). The mediating effect of digital literacy on the relationship between smartphone use motives and life satisfaction for senior citizens in Korea. Iran J Public Health.

